# Variation of natural selection in the Amoebozoa reveals heterogeneity across the phylogeny and adaptive evolution in diverse lineages

**DOI:** 10.3389/fevo.2022.851816

**Published:** 2022-08-04

**Authors:** Fang Wang, Yonas I. Tekle

**Affiliations:** Department of Biology, Spelman College, Atlanta, GA, United States

**Keywords:** natural selection, purifying selection, adaptive evolution, codon usage bias, Amoebozoa

## Abstract

The evolution and diversity of the supergroup Amoebozoa is complex and poorly understood. The supergroup encompasses predominantly amoeboid lineages characterized by extreme diversity in phenotype, behavior and genetics. The study of natural selection, a driving force of diversification, within and among species of Amoebozoa will play a crucial role in understanding the evolution of the supergroup. In this study, we searched for traces of natural selection based on a set of highly conserved protein-coding genes in a phylogenetic framework from a broad sampling of amoebozoans. Using these genes, we estimated substitution rates and inferred patterns of selective pressure in lineages and sites with various models. We also examined the effect of selective pressure on codon usage bias and potential correlations with observed biological traits and habitat. Results showed large heterogeneity of selection across lineages of Amoebozoa, indicating potential species-specific optimization of adaptation to their diverse ecological environment. Overall, lineages in Tubulinea had undergone stronger purifying selection with higher average substitution rates compared to Discosea and Evosea. Evidence of adaptive evolution was observed in some representative lineages and in a gene (Rpl7a) within Evosea, suggesting potential innovation and beneficial mutations in these lineages. Our results revealed that members of the fast-evolving lineages, *Entamoeba* and Cutosea, all underwent strong purifying selection but had distinct patterns of codon usage bias. For the first time, this study revealed an overall pattern of natural selection across the phylogeny of Amoebozoa and provided significant implications on their distinctive evolutionary processes.

## Introduction

Genetic mutation is the basic cause of diversity among organisms. In population genetics, the fate of a mutation (whether fixed or lost) depends on the collective effect of natural selection and random genetic drift (Sung et al., 2012). Natural selection is an important evolutionary mechanism for shaping variation in populations by promoting beneficial mutations and removing deleterious ones. The strength of natural selection and how it shapes the patterns of variation depends on many factors. Among these, a notable factor is effective population size (*Ne*) which play a key role in the molecular evolution and variation (Bierne and Eyre-Walker, 2004). Natural selection is more efficient in species with large *Ne* while species with small *Ne* are subject to strong genetic drift and are more prone to accumulate slightly deleterious mutations (Ingvarsson, 2010; Raynes et al., 2018). Effective population size together with other factors such as models of selection and patterns of linkage are related with rates of adaptive divergence in different species (Bachtrog, 2008; Strasburg et al., 2011). Studies on patterns of natural selection are essential in understanding the molecular evolution and gene functions of protein-coding genes within and among species (Bierne and Eyre-Walker, 2004; Flowers et al., 2012; De La Torre et al., 2017).

The strength of natural selection on protein-coding genes can be measured by the rates of non-synonymous substitutions (dN) to synonymous substitutions (dS). The values of dN/dS, denoted as omega (ω), could indicate positive selection (ω *>* 1), neutral evolution (ω = 1), and purifying selection (ω *<* 1) (Yang and Nielsent, 2002). Purifying selection would reduce genetic diversity by removing deleterious mutations due to the structural and functional constraint of genes. The speed of this mechanism depends on *Ne* and generation time of species (Sung et al., 2012). Genetic diversity at linked neutral sites is affected by purifying selection as found in various organisms (e.g., Flowers et al., 2012; Comeron, 2014; Elyashiv et al., 2016). Adaptive evolution driven by natural selection will increase the occurrence of beneficial traits in a population, and its rate depends largely on *Ne* (Bierne and Eyre-Walker, 2004; Strasburg et al., 2011). Moreover, natural selection can also affect codon usage bias (CUB) – an indicator of gene expression by optimization of transcription and translation (Hershberg and Petrov, 2008; Zhou et al., 2016; Galtier et al., 2018).

The supergroup Amoebozoa is a monophyletic clade comprising various amoeboid life forms of diverse morphology, ecology, behavior, life cycle, genome sizes, and complexity (Tekle et al., 2016, 2017, 2022; Kang et al., 2017). Most recent phylogenomic study generally recognize three major clades (Tubulinea, Evosea, and Discosea) within the supergroup (Kang et al., 2017). However, the deep level relationship among these major subclades and placement of some enigmatic lineages remains controversial. One of the main challenges in molecular phylogenetics of Amoebozoa has been the observed variation in molecular evolution of its members (Tekle et al., 2008).

Amoebozoa encompasses several fast evolving lineages known as long-branch taxa (LBT), whose phylogenetic positions have been difficult to determine in the tree of Amoebozoa (Tekle et al., 2008; Kang et al., 2017; Lahr et al., 2019). Particularly, LBT include both parasitic (e.g., *Entamoeba*) and several free-living lineages scattered throughout the major clades of Amoebozoa (e.g., Cutosea, *Stygamoeba, Parvamoeba*, and *Trichosphaerium*) (Tekle et al., 2008; Kang et al., 2017). Although it is well recognized that parasitic mode of life is associated with rapid evolution and adaptation coupled with large population size and short generation time due to the co-evolutionary arms race of host-parasite interaction (Das and Ganguly, 2014; Papkou et al., 2016), the Entamoebidae parasites within Evosea are rarely studied in this regard. *Dictyostelium discoideum*, a well-studied model amoeba, shows high estimates of population recombination and large *Ne* with low mutation rate (Flowers et al., 2010; Kucukyildirim et al., 2020). However, the forces driving the high genetic variations observed in the majority of amoebozoans are not investigated at a population or species level within a phylogenetic framework. The extreme diversity observed in Amoebozoa poses critical questions as to whether there are any patterns in their selection pressure and how this could give insights into the nature of adaptive evolution in this supergroup. While each lineage might have evolved under multiple driving forces, investigation on the variation of selection pressure across the Amoebozoa within a phylogenetic framework can provide important insights into their evolutionary process and help interpret the molecular basis of special behaviors of a particular lineage or groups.

In this study, we used highly conserved protein-coding genes that have been used for phylogenetics and estimation of species divergence time in previous studies (Parfrey et al., 2011; Kang et al., 2017). These orthologous genes were collected from a broad sampling across Amoebozoa. Based on these datasets, we employed codon-based substitution models in the program codeml (a package of PAML – Phylogenetic Analysis by Maximum Likelihood) to estimate the strength of natural selection among lineages (branch models) and sites (site models) using a phylogenetic framework (Yang, 2007). With this framework, we compared patterns of selective pressure among different levels of subgroups in Amoebozoa and investigated possible correlations to environmental factors and biological traits across species and clades within various groups. Furthermore, we analyzed CUB of these genes among Amoebozoa lineages to assess the effect of selection on their molecular evolution. CUB is the unequal frequencies of synonymous codons and patterns of CUB mainly depend on mutation and natural selection (Hershberg and Petrov, 2008; Plotkin and Kudla, 2011).

## Materials and methods

### Preparation of dataset

The dataset included selected genes from transcriptomes and genomes of major clades (Discosea, Evosea, and Tubulinea) in Amoebozoa (Supplementary Tables 1, 2). A total of 81 species of Amoebozoa (Supplementary Table 2) and one outgroup *Homo sapiens* (GRCh38.p13) were used in this study. Transcriptome assembly was performed as described in Tekle and Wood (2018). Originally, the gene pool consisted of 332 genes free of paralogs obtained from previous studies (Parfrey et al., 2011; Kang et al., 2017) (Supplementary Table 1). Program tblastn (Altschul et al., 1990) was used to obtain the sequences from each transcriptome/genome using 332 reference genes with e-value set at 1e-15 and the best hit was chosen for each species. The final alignments were checked against the reference genes manually to ensure good match and that no paralogs were included in final analysis. Program MACSE was used to align all 332 protein-coding sequences while also keeping the codon frame (Ranwez et al., 2018). Trimal v1.4 (Capella-Gutiérrez et al., 2009) was used to trim the alignments by removing poorly aligned regions with automatic method based on similarity statistics. Sites with over 50% gaps were removed in all the sequences. Sequences with over 50% gaps were then treated as incomplete and removed. The alignments were finally checked manually for confirmation.

To select genes that qualify for this study, model M0 (one ratio; NSsites = 0, model = 0) in the program codeml (PAML) (Yang, 2007) was performed for genes in each clade with more than 6 sequences. Model M0 assumes an identical ω among all branches and sites. Codon frequency option was set as F3×4 (Goldman and Yang, 1994). The tree topology used for each gene was generated using RAxML with GTRGAMMA option (Stamatakis, 2014). Based on the results of M0 model, genes with any branch that had dS or dN *>* 3 (indicating saturation of substitutions), or dS *<* 0.01 (leading to inaccurate estimates) were abandoned. The analysis was repeated twice and the result with larger log likelihood score was retained. The same procedure was adopted in all other models. The qualified genes from each major clade were kept for selective pressure studies with various models in codeml. The models used for selective pressure and corresponding datasets were summarized in Table 1.

### Estimation of the variation in selective pressures among branches across the whole phylogeny of Amoebozoa

To check the selective pressures (ω) across lineages in the phylogeny of Amoebozoa, branch model M1 (free-ratio; NSsites = 0, model = 1) in codeml (Yang, 2007) was applied to the concatenated shared genes among Discosea, Evosea, and Tubulinea including the outgroup *H. sapiens*. This model computes ω, dN, and dS values for all the branches in the phylogeny and allows for heterogeneity. Histogram and kernel density plots were used to visualize the distribution of each parameter in each major clade and plotted using function hist and density in R (R Core Team, 2014). Parameters of ω, dN and dS were compared between each of the two major clades using two-sample *T*-test (Fay and Proschan, 2010). Tree topology for M1 was from previous phylogenomics study with updated relationship within Tubulinea (Kang et al., 2017; Lahr et al., 2019). M1 test was performed with branch length inferred from the corresponding M0 model and set as initial values (fix_blength = 1). To test the effect of tree topology on selective pressures among branches, we performed M1 model based on an alternative tree topology where Tubulinea was distantly related with Discosea and Evosea (Tekle et al., 2022).

### Estimation of selective pressure within Discosea, Evosea, and Tubulinea using branch model

To test whether species evolve under different selective pressures within different major clades (Discosea, Evosea, and Tubulinea), we used the 2-ratio branch model (M2, model = 2) and tested each species separately. All suitable genes were included for each major clade and the corresponding tree topology was extracted from the whole tree using function getMRCA in R. This test was achieved by setting each of the species as a foreground branch, and the remaining branches in the phylogeny as background. Comparisons of 2-ratio test with M0 test were performed using likelihood ratio tests (LRT) to check for significance of different ω in each species (Yang, 1998). False discovery rate (FDR) correction was applied for multiple testing using *q*-value package from R with significance level of 0.05 (Korthauer et al., 2019). 2-ratio model was compared against the null model (fix_omega = 1, omega = 1) for tests of positive selection in those species that presented significantly different ω. LRT was performed using function pchisq in R.

To estimate the variation of selective pressures among branches in the two subclades (Centramoebia and Flabellinia) within Discosea, several hypotheses were investigated using branch models in codeml. H0: the two subclades have identical ω for all the lineages (model M0); H1: different ω in the branch leading to subclade Centramoebia (model M2); H2: different ω in the branch leading to subclade Flabellinia (model M2); H3: unique ω in the branch leading to Centramoebia and Flabellinia (model M2); H4: all branches have unique ω (model M1). The likelihood scores from each hypothesis were then compared for inference.

### Detection of episodic positive selection in the stem branches with branch-site model

**FIGURE 1:**
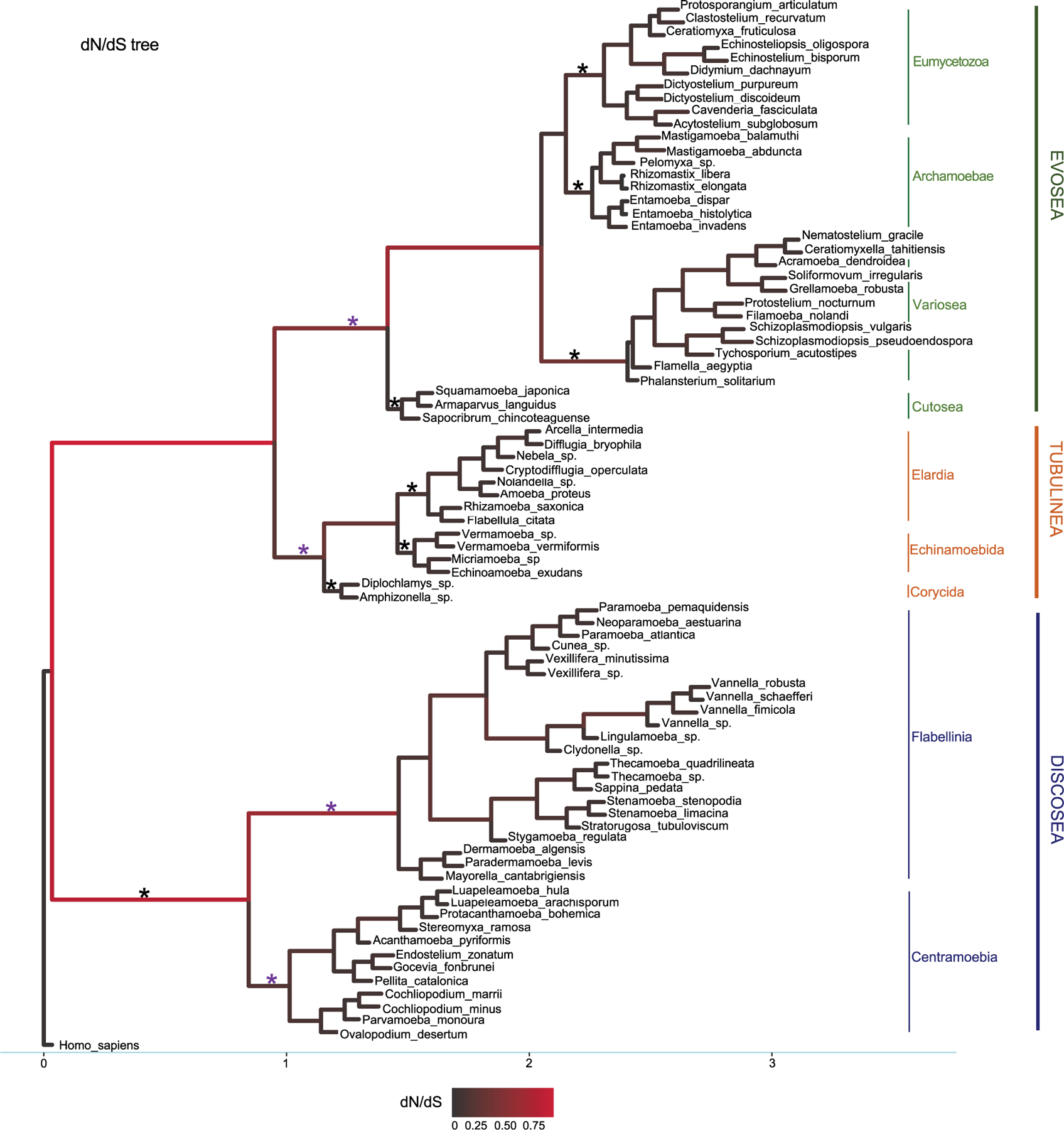
Phylogeny used in the M1 model with dN/dS values as branch lengths for visualization. The dataset is from the concatenated 20 shared genes comprising 10,365 sites. Subclade names were marked accordingly. Branches were colored by dN/dS values. Branches tested in branch-site model were marked with star symbols and purple star represented detection of positive selected sites.

To detect sites under positive selection in particular branches, branch-site model was performed based on the concatenated shared genes. We tested stem lineages including branches leading to major clades Discosea, Evosea, and Tubulinea, subclades Centramoebia and Flabellinia within Discosea and subclades Archamoebae, Eumycetozoa, and Cutosea within Evosea (branches tested were starred in Figure 1). Each of these lineages were set as the foreground branch alternately to have a unique ω distribution and was allowed to have proportion of sites under positive selection. All of the rest background branches in the phylogeny were allowed to share the same distribution of ω among sites. The branch-site model was specified by setting model = 2, NSsites = 2. Whole tree topology was used in this analysis. Branch lengths from M0 model were used as initial values (fix_blength = 1) to start the iteration for the alternative model. All analyses were repeated twice and the result with larger likelihood score was retained. LRT was conducted to determine significance of positive selection by comparing against the null model where foreground branch was set to have different proportion of sites under neutral selection but no positive selection. Same analysis was performed using the alternative tree topology as in M1 model.

### Estimation of substitution parameters and detection of positive selection across sites using site models

To estimate the variation of ω among sites and compare their distribution among major clades (Discosea, Evosea, and Tubulinea) in Amoebozoa, site models in codeml were applied in each of the major clade alternatively using concatenated shared genes (Yang et al., 2000). Tree topology used for each major clade was extracted from the whole phylogeny as above. Site models included M0 (one ratio), M1a (Nearly Neutral), M2a (Positive Selection), M3 (discrete), M7 (beta), M8 (beta&ω), and M8a (beta&ωs = 1); and were realized by setting the parameter NSsites in the control file (model = 0, NSsites = 0 1 2 3 7 8) (Yang et al., 2000; Yang, 2007). The site models allow ω ratio to vary in different patterns among codon sites. Specifically, M2a, M3, and M8 allow the presence of positively selected sites. The significance of evidence of positive selection was tested by comparing three pairs of models (M2a–M1a, M8–M7, and M8–M8a). LRT were used to compare different models based on log-likelihood scores. Comparison M3–M0 was performed for significance of ω variation among sites using LRT (df = 4). Positively selected sites from M2a and M8 were identified from Bayes Empirical Bayes (BEB) with posterior probabilities (Yang et al., 2005). Alternatively, we estimated the substitution parameters and signals of positive selection across sites in main subclades within Discosea and Evosea. Two subclades (Centramoebia and Flabellinia) within Discosea and three subclades (Archamoebae, Eumycetozoa, and Variosea) within Evosea were tested. Datasets included all suitable genes identified in each major clade.

### Estimation of substitution parameters for each gene in each major subclade

We had a systematic estimation of ω distribution and detection of sites under positive selection for each of the suitable genes in Discosea, Evosea, and Tubulinea using site models (M0, M1a, M2a, and M3). Statistics of the ω were checked in terms of range and mean. The difference of ω between each two major clades were compared using two-sample *T*-test. Comparison M2a–M1a and LRT were used for detection of positive selection across sites in each gene. Tree topology was generated using Raxml with GTRGAMMA option for each gene.

### Estimation of codon usage bias and correlations with selective pressure

Variables in codon usage bias (CUB) were estimated using CodonW (version 1.4^1^), including codon adaptation index (CAI), frequency of optimal codons (Fop), the effective number of codons (NC), G+C content (GC) and G+C content at 3rd codon positions (GC3s). CAI ranges from 0 (when a gene always uses the least frequently used synonymous codons) to 1 (when it always uses the most frequently used synonymous codons). Fop is the ratio of optimal codons to synonymous codons. NC measures the degree of codon usage in a gene and the value is between 20 (when only one codon is effectively used) and 61 (when codons are used randomly). Pearson’s correlation test was performed for correlation between CUB variables and ω using function cor.test in R.

## Results

### Selection of suitable genes

We started with 332 highly conserved orthologous genes, previously used in phylogenomic study of Amoebozoa (Kang et al., 2017) and divergence times in eukaryotes (Parfrey et al., 2011) to study patterns of selective pressure (Supplementary Table 1). We excluded genes that presented high substitution rates along any branch (dS or dN *>* 3), which is indicative of saturation of substitutions and genes with very low dS (*<*0.01) that could lead to inaccurate estimates (Yang et al., 2000). Most of the genes failed the criteria with high levels of saturation in synonymous substitutions in each clade (Supplementary Figure 1). The final dataset of suitable genes comprised only a small fraction – 35 genes in 34 species of Discosea, 43 genes in 33 species of Evosea, and 22 genes in 14 species of Tubulinea (Supplementary Tables 1, 2). A total of 20 shared genes were collected among these three major clades, which were used for analysis of the whole supergroup. The majority of these genes encodes ribosomal proteins (Supplementary Table 3), which is consistent with previous phylogeny study where non-ribosomal proteins showed larger saturation levels than ribosomal proteins (Cavalier-Smith et al., 2015).

### Estimation of variation in selective pressures among branches in the Amoebozoa

Using the concatenated 20 genes comprising 10,365 sites, we performed branch model M1 (free ratio) in codeml to check the variation of selective pressures among branches in the phylogeny of Amoebozoa with outgroup (Supplementary Figure 2).

M1 model allows different ω for each branch in the phylogeny. Results showed that all branches across the phylogeny were under purifying selection (ω *<* 1) (Figure 1). Statistics of substitution parameters (ω, dN, and dS) were checked (including mean and range for each clade) and compared among the three major clades (Table 2). *T*-test of pairwise comparisons (Discosea–Evosea, Discosea–Tubulinea, and Evosea–Tubulinea) showed no significant differences in any of the substitution parameters among the branches (*p*-values are 0.8125, 0.2474, and 0.3719, respectively). Histogram and kernel density plots of the substitution parameters indicate similar distributions among the three major clades (Supplementary Figure 3). While these clades displayed similarity in the overall distribution of these parameters, branches in Tubulinea had much smaller range of ω (0.0521–0.3023) compared to Discosea (0.0408–0.6180) and Evosea (0.0075–0.6334) (Table 2). The mean ω for all branches across the phylogeny of Amoebozoa was 0.1113. The mean and range of dN were similar for Discosea (0.0511, 0.0086–0.1549) and Evosea (0.0508, 0.0002–0.1594). The mean of dS in Tubulinea (0.8589) was relatively larger than that of Discosea (0.7247) and Evosea (0.6260), while the range was smaller (Table 2). The alternative tree topology where Tubulinea is the sister group to Discosea and Evosea did not change the results significantly and Tubulinea had different patterns than Discosea and Evosea (Supplementary Table 4).

Comparison of selective pressures showed differences among terminal branches (species) and internal branches in terms of ω values. Overall, terminal branches had smaller ω (mean ω = 0.0707) than those of internal branches (mean ω = 0.1528). Noticeably, several internal branches in Discosea and Evosea had larger ω (Figure 1) that resulted from relatively smaller dS values (Supplementary Figures 4, 5). These include branches leading to Flabellinia (ω = 0.618) within the Discosea, and Variosea (ω = 0.3549) within the Evosea (Figure 1).

We next looked specifically into parameters of the terminal lineages and checked patterns among the three major clades. The ω was 0.0075–0.1327 with a mean value of 0.0707 across all terminal lineages. *T*-test indicated no significant differences in ω for each paired comparison (Discosea–Evosea, Discosea–Tubulinea, and Evosea–Tubulinea, *p*-values were 0.0674, 0.1665, and 0.3895, respectively). No significant difference in terminal lineages was observed regarding dN in Discosea (0.0697), Evosea (0.0647), and Tubulinea (0.0860). The dS distribution in the species of each major clade revealed that Evosea (0.8143) had a much smaller mean value than Discosea (1.1081) and Tubulinea (1.2276) (Supplementary Table 5). Both Discosea–Evosea and Tubulinea–Evosea showed significant differences in the dS of the terminal lineages (*p*-values were 0.0418 and 0.0394, respectively). Overall, most of the species (terminal lineages) had smaller ω across the phylogeny, however, some species exhibited quite large dN or dS values (Supplementary Figures 4, 5 and Supplementary Table 5). Species that exhibited large dN include *Parvamoeba monoura, Stratorugosa tubuloviscum,* and *Planopodium desertum* within Discosea; *Sapocribrum chincoteaguense, Pelomyxa* sp., and *Mastigamoeba abducta* within Evosea; *Flabellula citata,* and *Micriamoeba* sp. within Tubulinea. Species that had large dS include *P. monoura*, *S. tubuloviscum,* and *Clydonella* sp. within Discosea, *S. chincoteaguense* within Evosea, and *F. citata,* and *Nolandella* sp. within Tubulinea (Supplementary Table 5).

The three species of the subclade Cutosea (*Armaparvus languidus*, *Squamamoeba japonica*, and *S. chincoteaguense*), which are among the fastest evolving lineages (Cavalier-Smith et al., 2016), demonstrated high synonymous and non-synonymous substitution rates with regard to their respective nodes, and had very small ω (Figure 1, Supplementary Figures 4, 5, and Supplementary Table 5), indicating strong purifying selection. The same case was also observed in the internal branch leading to the clade Entamoebidae with three parasitic species (*Entamoeba histolytica*, *Entamoeba dispar*, and *Entamoeba invadens*) within Evosea (Figure 1, Supplementary Figures 4, 5, and Supplementary Table 5).

### Detection of positive selection in branches with branch-site model

We used branch-site model to test episodic positive selection in selected lineages leading to major clades and subclades in the Amoebozoa using the concatenated alignments. This analysis included lineages leading to Discosea, Evosea, and Tubulinea and major subclades within each of these major clades (branches tested were starred in Figure 1). Significant results from likelihood ratio test (LRT) were observed in lineages leading to Evosea and Tubulinea as well as subclades Centramoebia and Flabellinia within Discosea (Table 3). From BEB analysis, the number of sites potentially under positive selection in these lineages ranged from 22 to 132 with a posterior probability over 50%; the number was from 1 to 62 with a probability over 95% (Table 3). The ω values for site classes 2a and 2b in the foreground lineages leading to Evosea and Tubulinea were estimated as infinity (ω = 999). This was due to the few synonymous changes that causes inaccurate estimates of ω. However, the LRT in this case were not affected and still reliable (Nozawa et al., 2009). Results from the alternative tree topology suggest differences in several branches (Supplementary Table 6). Positive selected sites were detected in more branches including branches leading to Discosea and Variosea.

### Estimation of ω distribution and detection of positive selection across concatenated genes using site models

Using the alignments of concatenated 20 shared genes, we applied a series of site models on each of the three major clades (Discosea, Evosea, and Tubulinea) separately (Supplementary Table 7). In this analysis we aimed to compare the distribution of ω across the alignments among the three clades and checked signals of sites under potential positive selection. In general, conserved sites (ω *<* 1) dominated all the clades, followed by neutral sites (ω = 1) and very few positively selected sites (ω *>* 1).

Model M8 (beta&ω) showed the best fit in each major clade, which assumed 11 categories of ω with 10 ω (0 *<* ω *<* 1) categories following a beta-distribution plus an additional ω category allowing positive selection (ω ≥ 1). Proportion of positively selected sites were only detected in model M8 in Tubulinea (ω = 7.67, 0.210%). Discosea and Evosea presented nearly identical parameter estimates from M8 and M8a (beta&ωs = 1) (Supplementary Table 7), indicating no positively selected sites. The proportion of neutral sites for Discosea and Evosea were 0.224 and 0.351%, respectively. Model M0 (One ratio) had the worst fit which assumed an identical ω among all branches and sites. M1a (Nearly Neutral) and M2a (Positive Selection) also had poor fit and showed identical estimates with a small proportion of sites under neutral selection and no sites positively selected (Supplementary Table 7). With the better-fitted models, M7, M8, and M8a, the average of ω among sites in alignments of Discosea, Evosea, and Tubulinea were 0.0778, 0.1104, and 0.0434, respectively. This suggested that Tubulinea taxa had much smaller chance of fixing non-synonymous mutations than synonymous mutations in the concatenated alignments and underwent stronger purifying selection than Discosea and Evosea.

Comparison among models suggested more details in the distribution of ω among sites. M3 (discrete)–M0 comparison showed great significance (*p*-value = 0) with variable ω among sites in all three major clades in Amoebozoa (Table 4). Comparisons M2a (Positive Selection)–M1a (Nearly Neutral), M8 (beta&ω)–M7 (beta), and M8–M8a were used to detect positive selection with LRT. For all the three major clades, only comparison M8–M7 was significant (*p <* 0.05, Table 4). A very small portion of positively selected sites were detected from BEB analysis in M2a or M8 (Supplementary Table 7). However, M8–M7 was prone to high false positives and comparisons M1a–M2a and M8–M8a were considered more stringent and powerful for positive detection (Yang et al., 2000), which were not significant in our results (Table 4). Consequently, our analyses demonstrated that no significant evidence of positively selected sites across the concatenated shared genes in any of the clade examined.

### Estimation of variation in selective pressure within Discosea, Evosea, and Tubulinea

In addition to the 20 shared genes examined above, we identified more suitable genes within each major clade and performed detailed selective pressure analyses. These analyses enabled us to further investigate variations of evolution among lineages within these clades with increased detection power (Yang et al., 2000). Below we presented results from each clade.

#### Discosea

The dataset of Discosea comprised 35 genes (18,186 sites) from a total of 33 species. We first used branch models to estimate the variation of selective pressure in the branches across the phylogeny of Discosea. To do this, we tested several hypotheses and compared their likelihoods (see Section “Materials and methods”). Hypothesis H4, that assumes all the branches had their own selective pressure across the phylogeny of Discosea, fitted the data best with the largest likelihood score. Omega in this analysis was 0.0301–0.1243 (Table 5). Hypothesis H0, which assumes identical ω across all branches, had the worst fit. Alternatively, we use 2-ratio branch model to test the variance of ω among each terminal lineage (branch leading to each taxon) in Discosea. Twenty six out of 33 species showed significant differences when set as a foreground branch and compared to the rest of the branches in Discosea (Supplementary Table 8). Comparison of the models for these 26 species against the null model, where ω were fixed to 1, showed that their ω were significantly different and smaller than 1, indicating no positive selection in these lineages.

We used site models to investigate the ω distribution across the concatenated alignments (35 genes) for subclades Centramoebia and Flabellinia, which had good taxonomic representations. Like the result of the whole clade of Discosea with concatenated 20 shared genes, the M8 model fitted best, followed by M8a, M7, and M3. Based on the better fitted models, ω was 0.0227–0.0306 in Centramoebia and 0.0586–0.0593 in Flabellinia. This result indicated a smaller chance in fixation of non-synonymous mutations compared to synonymous mutations and thus a stronger constraint in the molecular changes of the studied genes in Centramoebia than in Flabellinia. The majority of the sites were highly conserved in the alignments of Centramoebia and Flabellinia. Positively selected sites (ω = 2.556, 0.35%) were only detected in Centramoebia in M8. LRT results of M3–M0 support heterogeneity of ω among sites for both two subclades with significance (Supplementary Table 9). Evidence for positive selection from the three pairs of comparisons showed significance in M8–M7 in Centramoebia and Flabellinia and M8–M8a in Centramoebia. However, LRT for the most stringent M2a–M1a comparison was not significant in both Centramoebia and Flabellinia (Supplementary Table 9).

#### Evosea

The dataset of Evosea comprised 43 genes (22,089 sites) from a total of 33 species. Most of the species (30/33) showed significant different ω when set as a foreground and compared against the rest of the branches in the phylogeny of Evosea examined in 2-ratio model. None of these 33 species underwent positive selection when checked against a null model where ω was fixed to 1 (Supplementary Table 8).

Site model analyses were conducted in three subclades: Archamoebae, Eumycetozoa, and Variosea. The best fitted model was M3 in Archamoebae and M8 in Eumycetozoa and Variosea. Based on the better fitted models (M3, M7, M8, and M8a), the average ω were 0.0997, 0.2367, and 0.1111, respectively (Supplementary Table 9). A proportion of selected sites were detected in M8 for Archamoebae (ω = 285.9, 0.023%), Eumycetozoa (ω = 73.34, 0.796%), and Variosea (ω = 1.126, 3.12%). Though LRT comparison showed significance in M8–M7 for all subclades and also M8–M8a for Variosea, M2a and M1a all had identical estimates, suggesting no significant evidence of positively selected site in any of the subclades in Evosea (Supplementary Table 9).

#### Tubulinea

Based on 22 genes (11,640 sites) from 14 species in Tubulinea, 2-ratio branch model revealed that 7 species showed significant differences when allowed to have a unique ω than the rest of the lineages and none of them underwent positive selection (Supplementary Table 8). No subclades were checked in Tubulinea due to the small representations (less than 6) in most of the subclades.

### Estimation of selective pressure and detection of positive selection in individual genes

**FIGURE 2:**
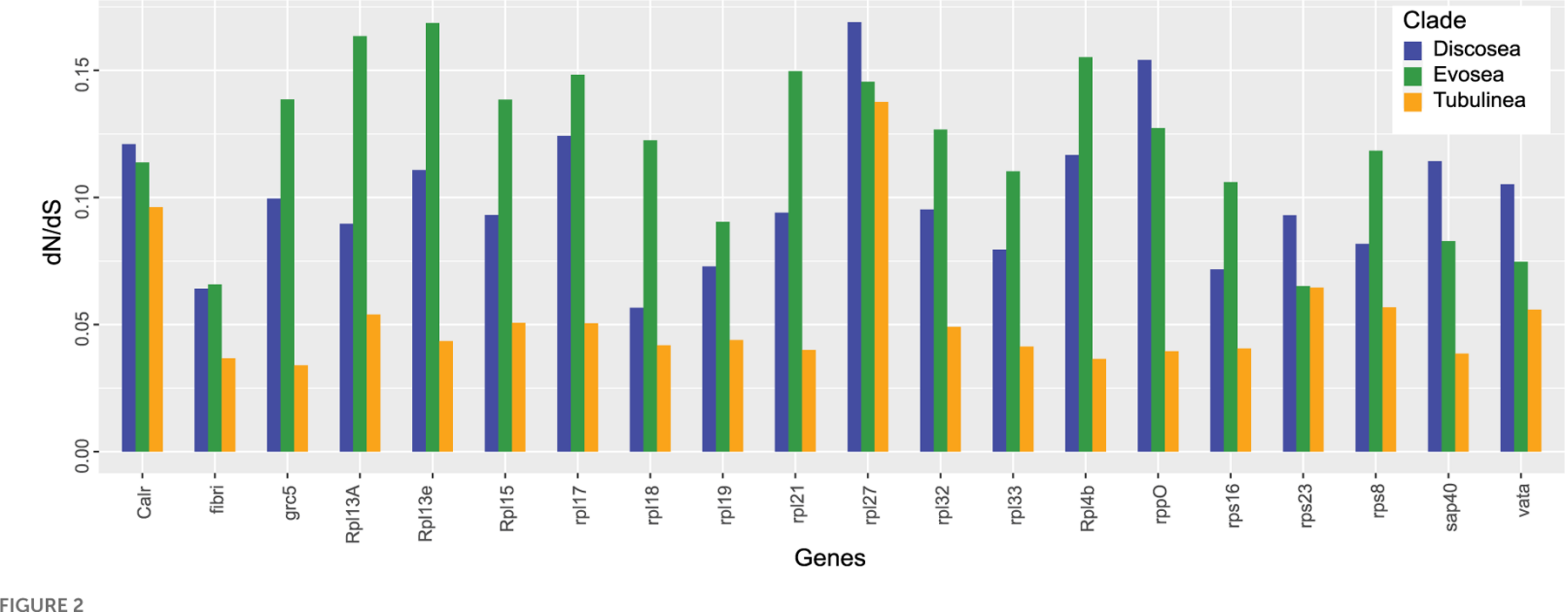
Estimates of dN/dS in each shared gene among Discosea, Evosea, and Tubulinea.

Results from site models (M0, M1a, M2a, and M3) supported that M3 fitted each gene best and ω of all the genes among the three major clades was 0.034–0.174 based on M3 (Supplementary Table 3). Moreover, LRT showed significance (*p*-value *<* 0.001) in comparison M3–M0, indicating three categories of selection fitted the data better than a global ω. The average ω among Discosea, Evosea, and Tubulinea were 0.098, 0.120, and 0.058, respectively. *T*-test showed significant differences (*p*-value *<* 0.05) in the ω of the genes between any of the two clades. *P*-values for Discosea–Evosea, Discosea–Tubulinea, and Evosea–Tubulinea were 0.0013, 2.353e-6, and 1.105e-10, respectively. Comparison of ω in the 20 shared genes exhibited large heterogeneity among Discosea, Evosea, and Tubulinea (Figure 2). LRT results in comparison M2a–M1a revealed that one gene (Rpl7a) in Evosea had significant evidence of positively selected sites and a proportion of 1.6% (ω = 4.13) were detected based on M2a. No positive selection across sites were detected in any of the genes examined in Discosea and Tubulinea.

### Correlations with codon usage bias and biological traits

**FIGURE 3:**
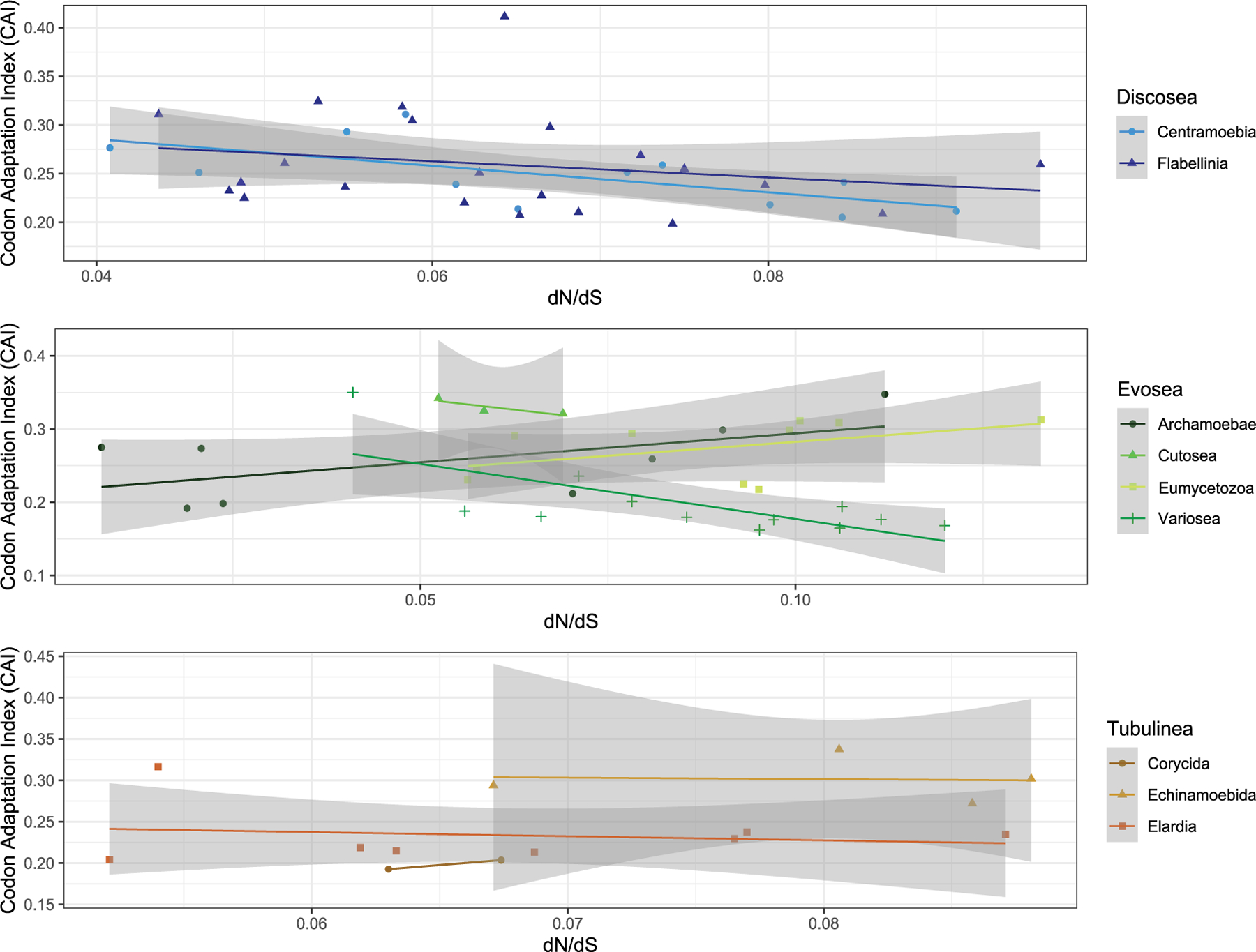
Correlation of dN/dS and Codon Adaptation Index in different subclades of Amoebozoa.

We assessed the correlation of selective pressure with codon usage bias (CUB) in species of Amoebozoa using the shared genes to understand the effect of natural selection and patterns of molecular evolution. Taking all of amoebae taxa into consideration, we observed a negative and non-significant correlation between ω and each of the CUB variables (CAI, Fop, Nc, GC, and GC3, see Section “Materials and methods”) (Supplementary Table 10). A significant positive correlation was observed between CAI and GC content (*p*-value = 2.88e-08, cor = 0.57), and also between CAI and GC content at the third codon position (*p*-value = 2.04e-07, cor = 0.54). Particularly, we investigated the correlation of CAI (Codon Adaptation Index) with ω and found different patterns in different subclades (Figure 3). CAI measures the relative adaptiveness of codon usage in a gene to that of the most abundant codon, and is associated with expression level – high CAI implying high expression (Jansen et al., 2003; Zhou et al., 2016). Subclades of Discosea all showed negative correlations between CAI and ω, while different patterns were found for subclades of Evosea and Tubulinea (Figure 3). CAI was 0.16–0.41 with an average of 0.25 across all species in Amoebozoa. The average CAI for species in Discosea, Evosea, and Tubulinea were quite similar (0.255, 0247, and 0.248, respectively). Among all subclades, Variosea (within Evosea) had the smallest CAI with an average of 0.18 (except for *Phalansterium solitarium* with 0.35), suggesting Variosea lineages tend to use the least frequently used synonymous codons. CAI and ω were negatively correlated with significance (*p*-value = 0.01, cor = −0.70) in Variosea (Figure 3).

We next investigated the long-branch taxa including three *Entamoeba* parasites (*E. histolytica, E. dispar,* and *E. invadens*) and three non-parasitic species in Cutosea (*A. languidus*, *S. japonica*, and *S. chincoteaguense*) for correlative purposes. The three *Entamoeba* parasites displayed small mean CAI (0.20). In contrast, the three Cutosea species had the largest mean CAI (0.33) among all subclades. Further inspection of all the 5 CUB variables revealed that *E. invadens* behaved differently from *E. histolytica* and *E. dispar* (Supplementary Table 10). Correlation test between CAI and ω was positive in the three *Entamoeba* parasites (*p*-value = 0.15, cor = 0.97) and negative in the three Cutosea species (*p*-value = 0.32, cor = −0.88) (Figure 3), both with no significance.

## Discussion

### Strong purifying selection and genetic saturation in Amoebozoa

Our analyses demonstrated that all branches in the Amoebozoa underwent purifying selection (ω *<* 1) based on the results from branch model M1 (free-ratio). The ω for all branches had a mean value of 0.1113. These results indicated the selective constraints necessary to maintain the structure and function of the studied genes, which included highly conserved genes and most of them encoded ribosomal proteins. The generally larger ω in internal branches compared to terminal branches suggested that ancestral lineages have a higher chance of fixation of non-synonymous mutations than synonymous mutations and a lesser degree of selection constraints.

Comparison of selective pressures and substitution rates among the three major clades (Discosea, Evosea, and Tubulinea) revealed a different pattern in Tubulinea. In general, larger levels of heterogeneity in ω were found in lineages within Discosea and Evosea than within Tubulinea (Figure 1 and Supplementary Table 8). This might be attributed to the high diversity (morphology and behavior) observed in Discosea and Evosea and relatively conserved and limited diversity in Tubulinea (Kang et al., 2017). Members of the Tubulinea clade share a defining morphological feature, that is monoaxially streaming and cylindrical pseudopods (Smirnov et al., 2005). In contrast, both Discosea and Evosea encompass members of extreme morphological and behavioral diversity and both clades lack unifying characteristics (synapomorphies). Tubulinea lineages also had a smaller average ω compared to Discosea and Evosea. This result was consistent with the results across concatenated alignments from site models (Supplementary Table 7). Furthermore, Tubulinea lineages showed higher average dN and dS than those in Discosea and Evosea albeit not significant, suggesting higher evolutionary rates including faster rates in both non-synonymous and synonymous sites.

Smaller ω is indicative of stronger and more efficient purifying selection and the accumulation of genetic changes in non-synonymous sites is less than synonymous sites. The stronger purifying selection in Tubulinea indicates higher degree of selective constraint on the structure and function of the studied genes (Cvijović et al., 2018). In population genetics, the fate of mutations is affected by selection and random drift, which depends largely on the effective population size (*Ne*) – more efficient selection are likely to act on larger populations (McVicker et al., 2009; Elyashiv et al., 2016). Taking this into account one possible explanation of the stronger purifying selection might be due to the overall larger *Ne* in Tubulinea compared to Discosea and Evosea. However, *Ne* in these lineages is not well known and such observation requires further investigation.

Unlike the other organisms (De La Torre et al., 2017), species in Amoebozoa showed widespread genetic saturation across genes used in phylogeny construction based on dS values (dS *>* 3). Despite the species divergence across amoebae, the saturation levels of these genes might also be due to incomplete lineage sorting (Philippe et al., 2011), which would also affect the estimation of selection (Yang and Nielsent, 2002). Genetic saturation levels of genes were considered to mainly contribute to the non-phylogenetic signal (Philippe et al., 2011). Though non-phylogenetic signal is part of the gene content, large proportion of saturated genes would cause difficulty in accurate resolution of the phylogeny (Philippe et al., 2011). Although the monophyly of the Amoebozoa as a whole has never been questioned, the deep relationships and placement of several of its members within the group have been controversial (Cavalier-Smith et al., 2016; Tekle et al., 2016; Kang et al., 2017; Lahr et al., 2019). A phylogenomic study reported that Evosea and Tubulinea are sister clades despite the lack of any morphological or other shared defining features (Kang et al., 2017). In contrast to this, a more recent phylogenomic study showed a close relationship between Evosea and Discosea, both of which are shown to have similar pattern of evolution compared to Tubulinea (Tekle et al., 2022). Given these conflicting reports, future phylogenetic studies might need to consider incomplete lineage sorting, saturation level and molecular evolution features of genes as well as improved models of evolution in order to better resolve the deep relationships of Amoebozoa.

### Positive selection in lineages leading to clades

Positive selection is difficult to detect in branch test and the branch-site model performs better in detecting positive selection (Yang and Nielsent, 2002; Zhang et al., 2005) giving that positive selection could affect only a few sites and occur in an episodic manner (Zhang et al., 2005). With this consideration we employed the branch-site model to test episodic evolution in specific lineages in the Amoebozoa phylogeny that had potential clade-specific evolutionary features (Zhang et al., 2005) (Figure 1). Adaptive evolution had been detected in set of sites along particular lineages and used for inference of gene duplication in gene family evolution or detection of functional divergence (Yang and Nielsent, 2002; Bielawski and Yang, 2004; Travers et al., 2005). Within different species, signatures of positive selection were detected for inference of genetic basis of species or clade-specific features along unique lineages (Vallender and Lahn, 2004; Travers et al., 2005). In our analyses, four lineages showed sites under positive selection and suggested advantageous mutations in the corresponding subclades including Tubulinea, Evosea, Centramoebia, and Flabellinia (marked with purple star in Figure 1). Among these four clades Tubulinea is the only lineages with shared morphological, monoaxially streaming and cylindrical pseudopods, character. The remaining clade, Evosea, Centramoebia, Flabellinia, and Stygamoebida, encompass lineages of diverse morphology and ecology based on molecular analysis and have no well-known unifying features. Excess of non-synonymous substitutions in these main internal branches of the phylogeny indicates their fixation in the clade (Bush, 2001) and this result provides insights into the adaptation and evolution of potential clade-specific traits in these groups that can be investigated further.

Detection of sites under positive selection is a difficult statistical problem. Branch-site model is known to be sensitive and the power of it depends on many factors such as sequence length, number of lineages, and strength of positive selection (Wong et al., 2004). While we treated the alignments in a rigorous way, any ambiguous sites can result in false positive estimates of positive selection at specific sites (Wong et al., 2004). Moreover, results from this model can also be affected by tree topology (Diekmann and Pereira-Leal, 2016). Whether selection and adaptive process promoted innovation at different levels depends on several mechanisms such as mutation, recombination, and random genetic drift (Lynch et al., 2014). Our results provided important information on specific sites that might account for features of adaptation in corresponding clades and further analysis in population genetics would give more insights into the evolution of specific groups.

### Patterns of selective pressure in individual genes

Detailed investigation of each gene revealed that gene Rpl7a in Evosea showed evidence of positive selection. The alignment of Rpl7a in Evosea consisted of 702 sequence sites from 29 species. Three sites were detected with signal of positive selection and the posterior probability was over 85%. This gene is highly conserved and encodes 60S ribosomal protein L7a in eukaryotic cells with distinct sequence in the promoter region than other eukaryotic ribosomal protein genes (Colombo and Fried, 1992). Our result suggested advantageous mutations in Rpl7a in lineages of Evosea.

The majority of genes in our study encode subunits (60S and 40S) of ribosomal proteins. Ribosomal proteins normally have high degree of conservation and due to their essential role in ribosome assembly and protein translation, they have high expression level and slow evolutionary rates (Drummond et al., 2005). However, species-specific selective pressures were considered as a substantial way to optimize adaptation at all levels of genes (Yadav et al., 2016). In our results, the average ω among all ribosomal proteins among species in Amoebozoa was 0.105, which was quite similar to that of *Arabidopsis thaliana* and *Drosophila melanogaster* albeit more ribosomal proteins were involved in their study (Moutinho et al., 2019). Differential signatures of selection in each gene were observed in Discosea, Evosea, and Tubulinea, with Tubulinea showing the smallest ω in all of the studied genes. This could be a result of optimized adaptation for diverse environments in different clades. Moreover, heterogeneity in ω was observed in different ribosomal genes of each clade (Figure 2). This is consistent with previous study that ribosomal proteins were potentially under various selection for adaptation to different environmental conditions (Yadav et al., 2016). This process of adaptation involved possible optimized combination of ribosomal proteins with expression regulation (Yadav et al., 2016). Further study combined with the function and expression of these genes will provide more information on the evolution of ribosomal genes in each amoeba clade. It should be noted that some ribosomal genes were reported to be evolving under concerted evolution where multiple copies of rDNA within a species undergo genetic exchange (Ganley and Kobayashi, 2007). If this form of evolution was true for our ribosomal genes, the result in this study will be compromised. However, at the moment we cannot compare the two forms of evolution in these ribosomal genes due to the lack of complete genomes of most species and the repeat variation levels were unknown.

Protein RPL27L, a recent paralog of Rpl7a, was reported to have low expression compared to other core ribosomal proteins in mice and human (Yadav et al., 2016), showing a different response to selective pressure. Our result also showed that gene Rpl27 had a distinct level of selective pressure with the largest ω among all the studied genes in Amoebozoa, especially in Discosea (Figure 2). This finding suggested interesting pattern of evolutionary process of this gene that should be looking into further. This recently evolved paralog might still have the ongoing process of adaptation that induced more genetic changes in the non-synonymous sites (Kryazhimskiy and Plotkin, 2008).

### Patterns of molecular evolution in fast evolving parasitic and non-parasitic amoebozoans

Our results reveal complex evolutionary processes regardless of mode of life, morphological or behavioral difference in the Amoebozoa. We particularly examined results for some special subgroups including parasitic *Entamoeba* (*E. invadens*. *E. dispar,* and *E. histolytica*), and free-living marine Cutosea which are long-branch taxa with problematic phylogenetic position in the tree of Amoebozoa (Cavalier-Smith et al., 2016; Tekle et al., 2016; Kang et al., 2017). We also assessed results in Variosea, which are characterized with distinct morphotypes (Berney et al., 2015). These comparisons give a glimpse of the complex evolutionary pattern of the group in general and possible factors influencing their selective pressure and codon usage bias.

There were few studies on the population genetics of *Entamoeba* (e.g., Das and Ganguly, 2014). Parasites are generally prone to elevated rates of evolution, due to their short generation times and large *Ne* (Kochin et al., 2010; Watson, 2013). If this is true for *Entamoeba*, stronger purifying selection is expected in the group due to forces of natural selection tend to be more efficient in larger populations (Raynes et al., 2018). Our results supported this theory where a much stronger purifying selection was observed in parasitic *Entamoeba* than in most of non-parasitic lineages of Amoebozoa. Within the genus *Entamoeba*, *E. dispar,* and *E. histolytica* showed huge similarity in selective pressure and codon usage, while they had large difference compared to *E. invadens* (Supplementary Table 10). Similar result on codon usage bias among these species was also previously reported based on different gene sets (Nozaki et al., 1997). Both *E. dispar* and *E. histolytica* reside in mammals and are morphologically indistinguishable (Das and Ganguly, 2014), while *E. invadens*, infects reptiles (Hooshyar et al., 2015) and is morphological and genetically distant. These observations suggest the importance of ecological environment in shaping their evolutionary processes. The observed variance in these parasitic *Entamoeba* species might be explained by the different adaptive response to the ecological niche in the host regardless of pathogenicity.

Among the free-living amoebozoans, Cutosea stands out in its pattern of evolution (small ω and strong purifying selection) similar to *Entamoeba.* Very little is known about Cutosea diversity and this novel lineage has only three representatives (Kudryavtsev and Pawlowski, 2013; Schuler and Brown, 2019). All these species are described from marine habitat and share unique cell coat of microscales that are separated from the cell membrane. A general trend of elevated genetic load and mutation rates has been reported in marine animals compared to terrestrial animals (Sauvage et al., 2007; Plough, 2016) and marine animals tend to have large *Ne*. We did not find in the literature reports that show similar correlation in unicellular eukaryotes, but the possibility of large *Ne* in Cutosea could explain the observed strong purifying selection and largest CAI in this group than others where population can respond to weak selection and effective translational selection on codon usage takes place (Ingvarsson, 2010; Galtier et al., 2018). The patterns of CUB mainly depend on mutation and natural selection (Hershberg and Petrov, 2008; Plotkin and Kudla, 2011). Particularly for unicellular organisms, mutational mechanisms is a major factor for interspecific variation in codon usage while selection explains more on variation across a gene or genome (Sharp et al., 2010; Plotkin and Kudla, 2011). This explains the diverse correlations between CAI and selective pressure in different subclades across amoebozoans with few of them significant indicating complex evolutionary forces influencing much of their mutations.

Particularly, we found that species in Cutosea showed elevated synonymous substitution rates which was also observed in other marine or salt water amoeba species including *P. monoura, Clydonella* sp., *F. citata,* and *Nolandella* sp. (Cole et al., 2010; Kudryavtsev et al., 2011; Kudryavtsev and Pawlowski, 2013) (Supplementary Table 5). These amoebae are evolutionary diverse and represent the three major clades of Amoebozoa. In addition an interesting observation is the various types of cell coats observed in long-branch lineages including Cutosea, *Cochliopodium*, *Parvamoeba, Trichosphaerium,* and *Dermamoeba* (Cole et al., 2010; Kudryavtsev et al., 2011), which all appear to have a relatively elevated rates of evolution (Tekle et al., 2008; Kang et al., 2017). The ability to liberate plasma membrane through the intricate cell coats of these evolutionary diverse lineages and its correlation with the observed pattern of evolution is of interest to investigate. The evolution of cell coat and marine environment might be considered as potential features that shape the variation and evolutionary process in Cutosea.

Our results showed that Variosea lineages exhibited the smallest CAI and tended to use the least frequently used synonymous codons among all subclades. CAI could be a predictor for gene expression level largely through its effect on optimization of transcription and translation (Jansen et al., 2003; Zhou et al., 2016). Taking this assumption, the studied genes (mostly ribosomal protein coding genes) had the lowest expression level in Variosea than other subclades in Amoebozoa. This is an open question especially for lineages of this group which has diverse biological traits. Negative correlation is generally expected for evolutionary rate and gene expression level (Subramanian and Kumar, 2004; Park et al., 2012). However, high evolutionary rate is not obvious in Variosea. A significant negative correlation between CAI and ω was observed in Variosea, which supported the theory of stronger selection acting in highly expressed genes (Gout et al., 2010). Additionally, the similarity in the expression level of these genes (indicated by CAI) and selective pressure within Variosea is a surprising result due to the diversity of their biological traits. More information on the population genetics of this group is needed to further our understanding of the evolutionary processes of the Variosea.

### Potential limitations and future perspectives

In this study, the usage of dN/dS to estimate the strength of natural selection is based on the assumption that synonymous mutations are neutral. Though most synonymous mutations are considered as neutral, this is not always the case and synonymous mutations were shown to have variable fitness effects especially for highly expressed genes (Lebeuf-Taylor et al., 2019). It is not known at this point whether selection act at the synonymous sites of these genes, however, if this was the case, the tests of saturation level of synonymous mutations and codon usage bias need to be re-evaluated. In conclusion, our study investigated the variation of natural selection among lineages and sites across the phylogeny of Amoebozoa based on a set of highly conservative genes. These results provide insights on how natural selection affects the substitution rates and codon usage and the possible correlations with their biological traits and ecological environment among different subclades within Amoebozoa. The limitation of the study is in the number of genes due to the limited number of species and sequences currently available. The divergence of genes among amoebae species makes the identification of homologous genes difficult, which are subject to paralogy. In this study we used the genes previously well investigated and applied in phylogenetic studies. It should be noted that different genes might have different patterns of selective pressure. Though we started with 332 genes, the result of this study is mainly based on ribosomal genes and should be considered patterns reflective of these genes of the group. The detection power of the tests might be affected by this limitation as well, which would be affected by the strength of positive selection in highly conservative genes. This could also explain the strong purifying selection observed due to the high structural and functional constraint. Further analysis with more types of protein-coding genes coupled with their functions and expression level as well as more information in the field of population genetics in the Amoebozoa species will help for a more detailed understanding of the forces that shape the evolution and diversity in Amoebozoa.

## Supplementary Material

supplementary files SUPPLEMENTARY FIGURE 1
Plots of the maximum synonymous rates (dS) across the branches in the phylogeny of each gene in each major clade according to Supplementary Table 1. Genes that had dS *<* 3 were marked as red. From top to bottom, the major clades are Discosea, Evosea, and Tubulinea.
SUPPLEMENTARY FIGURE 2
Tree topology with all the species and outgroup included. The branch length of the tree was estimated from M0 model in codeml.
SUPPLEMENTARY FIGURE 3
Histogram and kernel density plots of the substitution parameters (ω, dN, and dS) across all the branches in each major clade based on the results of M1 model.
SUPPLEMENTARY FIGURE 4
Phylogeny using all the species in the M1 model with dN as branch length for visualization. Branches were colored by dN values.
SUPPLEMENTARY FIGURE 5
Phylogeny using all the species in the M1 model with dS as branch length for visualization. Branches were colored by dS values.
SUPPLEMENTARY TABLE 1
Information of the initial 332 genes for each major clade in the Amoebozoa.
SUPPLEMENTARY TABLE 2
Information of the taxa used in this study.
SUPPLEMENTARY TABLE 3
Details of qualified genes in each clade and ω estimates from M3 model for each gene in each clade.
SUPPLEMENTARY TABLE 4
Statistics of parameters based on the results of branch model M1 (free ratio) across all the branches in each major clade, with an alternative tree topology where Tubulinea branches basal to Discosea and Evosea [(Discosea + Evosea), Tubulinea].
SUPPLEMENTARY TABLE 5
Parameter estimates of each species (terminal branch) from M1 model.
SUPPLEMENTARY TABLE 6
Results of likelihood ratio tests and numbers of positively selected sites using branch-site models with an alternative tree topology where Tubulinea branches basal to Discosea and Evosea [(Discosea + Evosea), Tubulinea].
SUPPLEMENTARY TABLE 7
Parameter estimates and log-likelihood scores from each site model with concatenated genes for each major clade of Amoebozoa.
SUPPLEMENTARY TABLE 8
Results for each taxon from 2-ratio model and model comparisons.
SUPPLEMENTARY TABLE 9
Site model results and comparisons for subclades in Discosea and Evosea.
SUPPLEMENTARY TABLE 10
Parameter estimates on selection and codon usage bias for each species.

## Figures and Tables

**TABLE 1:** A summarization of the datasets and the corresponding models used.

Datasets	Models applied from program codeml
20 shared concatenated genes; all species	Model M0
20 shared concatenated genes; all species	Branch model M1
20 shared concatenated genes; each major subclade	Site models M0, M1a, M2a, M3, M7, M8, M8a
20 shared concatenated genes; all species	Branch-site model
22 Tubulinea genes; 14 Tubulinea species	2-ratio branch model. Site models M0, M1a, M2a, M3, M7, M8, M8a.
43 Evosea genes; 33 Evosea species	2-ratio branch model; Site models M0, M1a, M2a, M3, M7, M8, M8a.
35 Discosea genes; 33 Discosea species	2-ratio branch model; Site models M0, M1a, M2a, M3, M7, M8, M8a.
Subclades within Discosea;	Site models M0, M1a, M2a, M3, M7, M8, M8a.
Subclades within Evosea	Site models M0, M1a, M2a, M3, M7, M8, M8a.
Each gene; each major subclade	Site models M0, M1a, M2a, M3.

**TABLE 2:** Statistics of parameters based on the results of branch model M1 (free ratio) across all the branches in each major clade.

Parameter	dN/dS		dN		dS	

	Mean	Range	Mean	Range	Mean	Range
Discosea	0.1032	(0.0408, 0.6180)	0.0511	(0.0086, 0.1549)	0.7247	(0.0233, 2.6511)
Evosea	0.0997	(0.0075, 0.6334)	0.0508	(0.0002, 0.1594)	0.6260	(0.0159, 2.3074)
Tubulinea	0.0869	(0.0521, 0.3023)	0.0626	(0.0102, 0.1931)	0.8589	(0.0499, 2.2167)

**TABLE 3:** Results of likelihood ratio tests and numbers of positively selected sites from BEB using branch-site models.

Lineage leading to	lnL		^†^ *P*_value	^‡^ PSS(BEB)
	Alternative	Null		
Discosea	−498311.531776	−498311.550412	0.8469	25(2)
Evosea	−498269.487794	−498310.113393	0***	22(7)
Tubulinea	−498154.743065	−498335.557743	0***	132(62)
Centramoebia/Discosea	−498289.962835	−498304.361529	8.035942e-08***	42(1)
Flabellinia/Discosea	−498285.840495	−498315.039608	2.14273e-14***	35(8)
Eumycetozoa/Evosea	−498314.349759	−498314.330187	1	48(10)
Variosea/Evosea	−498314.349476	−498310.284088	1	26(4)
Cutosea/Evosea	−498314.349759	−498314.349759	1	67(9)
Archamoebae/Evosea	−498314.349760	−498314.349759	1	48(17)
Corycida/Tubulinea	−498314.349760	−498314.349759	1	82(18)
Echinamoebida/Tubulinea	−498314.349725	−498314.349760	0.9933245	32(9)
Elardia/Tubulinea	−498314.349759	−498276.628024	1	49(21)

† *P*_value was marked using NEJM (New England Journal of Medicine) style for different levels.

* *p*-value *<* 0.05;

** *p*-value *<* 0.01;

*** *p*-value *<* 0.001.

‡ PSS: the number of positive selection sites obtained from Bayes Empirical Bayes. The first number is the PSS with posterior probabilities *>* 50% and the second with posterior probabilities *>* 95%.

**TABLE 4:** Model comparisons of site models in Discosea, Evosea, and Tubulinea in Amoebozoa.

Clade	Model	df^†^	LRT(2*DL*)	*p*-value
Discosea	M2a – M1a	2	0	1
	M8 – M7	2	12.78832	0.0017**
	M8 – M8a	1	0	1
	M3 – M0	4	20371.13	0*
Evosea	M2a – M1a	2	0	1
	M8 – M7	2	18.20107	0.0001***
	M8 – M8a	1	9.2e-05	1
	M3 – M0	4	16686.45	0*
Tubulinea	M2a – M1a	2	0	1
	M8 – M7	2	8.826102	0.0121*
	M8 – M8a	1	0.002642	0.9590
	M3 – M0	4	6092.276	0***

† df represents degree of freedom used in the LRT test.

* *p*-value *<* 0.05;

** *p*-value *<* 0.01;

*** *p*-value *<* 0.001.

**TABLE 5:** Parameter estimates and likelihood scores from branch models for the variation of ω among branches within Discosea based on the concatenated genes.

Hypothesis	Model	Foreground	Background	Parameter estimates	lnL
H0	M0(one-ratio)	^†^ ω1 = ω2 = ω0		ω1 = ω2 = ω0 = 0.0447	−362818.893706
H1	M2 (2ratio)	ω1	ω2 = ω0	ω1 = 0.0369; ω2 = ω0 = 0.0470	−362785.512107
H2	M2 (2ratio)	ω2	ω1 = ω0	ω2 = 999; ω1 = ω0 = 0.0448	−362766.269417
H3	M2 (2ratio)	ω1, ω2	ω0	ω1 = 0.0420; ω2 = 0.0451; ω0 = 0.0563	−362766.299673
H4	^‡^ M1(free-ratio)	All unique		ω range: 0.0301–0.1243; ω mean: 0.0560	−362223.571136

† ω1 represented omega estimates in the stem lineage leading to subclade *Centramoebia*; ω2 represented omega estimates in the stem lineage leading to subclade *Flabellinia*; ω0 represented omega estimates in the background lineages.

‡ Detailed results of M1 can be found in Table 2 and Supplementary Table 5.

## Data Availability

The original contributions presented in this study are included in the article/Supplementary material, further inquiries can be directed to the corresponding author.

## Footnotes

1 http://codonw.sourceforge.net
