# Supplementary material for: Variation of natural selection in the Amoebozoa reveals heterogeneity across the phylogeny and adaptive evolution in diverse lineages
Source: Front Ecol Evol. Author manuscript; Available in PMC 2023 Mar 2. (PMC9980437; doi:10.3389/fevo.2022.851816)
Supplement: supplementary files — SUPPLEMENTARY FIGURE 1 Plots of the maximum synonymous rates (dS) across the branches in the phylogeny of each gene in each major clade according to Supplementary Table 1. Genes that had dS < 3 were marked as red. From top to bottom, the major clades are Discosea, Evosea, and Tubulinea. SUPPLEMENTARY FIGURE 2 Tree topology with all the species and outgroup included. The branch length of the tree was estimated from M0 model in codeml. SUPPLEMENTARY FIGURE 3 Histogram and kernel density plots of the substitution parameters (ω, dN, and dS) across all the branches in each major clade based on the results of M1 model. SUPPLEMENTARY FIGURE 4 Phylogeny using all the species in the M1 model with dN as branch length for visualization. Branches were colored by dN values. SUPPLEMENTARY FIGURE 5 Phylogeny using all the species in the M1 model with dS as branch length for visualization. Branches were colored by dS values. SUPPLEMENTARY TABLE 1 Information of the initial 332 genes for each major clade in the Amoebozoa. SUPPLEMENTARY TABLE 2 Information of the taxa used in this study. SUPPLEMENTARY TABLE 3 Details of qualified genes in each clade and ω estimates from M3 model for each gene in each clade. SUPPLEMENTARY TABLE 4 Statistics of parameters based on the results of branch model M1 (free ratio) across all the branches in each major clade, with an alternative tree topology where Tubulinea branches basal to Discosea and Evosea [(Discosea + Evosea), Tubulinea]. SUPPLEMENTARY TABLE 5 Parameter estimates of each species (terminal branch) from M1 model. SUPPLEMENTARY TABLE 6 Results of likelihood ratio tests and numbers of positively selected sites using branch-site models with an alternative tree topology where Tubulinea branches basal to Discosea and Evosea [(Discosea + Evosea), Tubulinea]. SUPPLEMENTARY TABLE 7 Parameter estimates and log-likelihood scores from each site model with concatenated genes for each major clade of Amoebozoa. SUPPLEMENTARY TABLE 8 Results for each ta [file NIHMS1871028-supplement-supplementary_files.zip › Data_Sheet_3_Variation of natural selection in the Amoebozoa reveals heterogeneity across the phylogeny and adaptive evolution in diverse lineages.ZIP!/Supplementary Tables.zip!/Table S7. SiteModels_TED.docx]

**Table S7.** Parameter estimates and log-likelihood scores from each site model with concatenated genes for each major clade of Amoebozoa.

| Clade | Model | No_seqs | No_sites | dn/ds | ^†^ncatG | Parameter Estimates | ^‡^PSS | ^§^ lnL |
| --- | --- | --- | --- | --- | --- | --- | --- | --- |
| DISCOSEA | M0 (one-ratio) | 34 | 11301 | 0.0568 | 1 | ω = 0.0568 | NA | -222916.635705 |
|  | M1a (NearlyNeutral) | 34 | 11301 | 0.1700 | 2 | p0 = 0.89095; p1 = 0.10905;  ω0 = 0.06844; ω1 = 1 | NA | -220097.507580 |
|  | M2a (PositiveSelection) | 34 | 11301 | 0.1700 | 3 | p0 = 0.89095; p1 = 0.10905; p2 = 0.00000;  ω0 = 0.06844; ω1 =1; ω2 = 1 | 0 | -220097.507580 |
|  | M3 (discrete) | 34 | 11301 | 0.0718 | 3 | p0 =0.40767; p1= 0.38229; p2 = 0.21004  ω0 =0.00394; ω1 = 0.06318; ω2 = 0.21928 | 0 | -212731.070309 |
|  | M7 (beta) | 34 | 11301 | 0.0779 | 10 | p = 0.40681; q = 4.59073 | NA | -212417.697851 |
|  | M8 (beta&ω) | 34 | 11301 | 0.0778 | 11 | p0 = 0.99776 p = 0.41269 q = 4.79613  (p1 = 0.00224) ω = 1.00000 | 6(2) | -212411.303689 |
|  | M8a (beta&ωs =1) | 34 | 11301 | 0.0778 | 11 | p0 = 0.99776 p = 0.41269 q = 4.79613  (p1 = 0.00224) ω = 1.00000 | NA | -212411.303689 |
| EVOSEA | M0 (one-ratio) | 33 | 10521 | 0.0863 | 1 | ω = 0.0863 | NA | -195196.186311 |
|  | M1a  (NearlyNeutral) | 33 | 10521 | 0.2189 | 2 | p0 = 0.85088; p1 = 0.14912;  ω0 = 0.08199; ω1 =1 | NA | -191805.481880 |
|  | M2a  (PositiveSelection) | 33 | 10521 | 0.2189 | 3 | p0 = 0.85088; p1=0.14912; p2=0.00000;  ω0 = 0.08199; ω1 =1; ω2= 1 | 4(0) | -191805.481880 |
|  | M3 (discrete) | 33 | 10521 | 0.1053 | 3 | p0 = 0.40935; p1= 0.40128; p2 = 0.18936;  ω0 = 0.00746; ω1 = 0.09689; ω2 = 0.33481 | 0 | -186852.958848 |
|  | M7 (beta) | 33 | 10521 | 0.1109 | 11 | p = 0.42437; q = 3. 27587 | NA | -186579.418938 |
|  | M8 (beta&ω) | 33 | 10521 | 0.1102 | 11 | p0 = 0.99649 p = 0.43293 q = 3. 47471  (p1 = 0.00351); ω = 1.00000 | 5(0) | -186570.318402 |
|  | M8a (beta&ωs =1) | 33 | 10521 | 0.1102 | 11 | p0 = 0.99224; p = 0.43285 q = 3. 47386  (p1 = 0.00351) ω = 1.00000 | NA | -186570.318448 |
| TUBULINEA | M0 (one-ratio) | 22 | 12408 | 0.0269 | 1 | ω = 0.0269 | NA | -96539.906889 |
|  | M1a  (NearlyNeutral) | 22 | 12408 | 0.0918 | 2 | p0 = 0.93236; p1 = 0.06764;  ω0 = 0.02594; ω1 =1 | NA | -95758.773662 |
|  | M2a  (PositiveSelection) | 22 | 12408 | 0.0918 | 3 | p0 = 0.93236; p1 = 0.02983; p2 = 0.03781;  ω0 = 0.02594; ω1 = 1; ω2=1 | 12(0) | -95758.773662 |
|  | M3 (discrete) | 22 | 12408 | 0.0366 | 3 | p0 = 0.38050; p1= 0.41226; p2 = 0.20724  ω0 = 0.00200; ω1 = 0.03140; ω2 = 0.11041 | 0 | -93493.768924 |
|  | M7 (beta) | 22 | 12408 | 0.0376 | 10 | p = 0.51909; q = 12.62820 | NA | -93483.870497 |
|  | M8 (beta&ω) | 22 | 12408 | 0.0524 | 11 | p0 = 0.99790; p = 0.52564; q = 13.22035  (p1 = 0.00210) ω = 7.67181 | 6(0) | -93479.457446 |
|  | M8a (beta&ωs =1) | 22 | 12408 | 0.0402 | 11 | p0 = 0.99565; p = 0.53140; q = 13.52975  (p1 = 0.00435) ω= 1 | NA | -93479.458767 |

^†^ ncatG: number of categories in the ω distribution for each site model.

^‡^ PSS: the number of positive selection sites obtained from BEB. The first number is the PSS with posterior probabilities > 50%. The second number (in parentheses) is the PSS with posterior probabilities > 95%.

^§^ lnL: the log likelihood estimation from each site model obtained from codeml (PAML) for each clade.

^¶^ Parameter estimates p0, p1, and p2 in models M1a and M2a, represent the proportion of conserved sites (ω < 1), neutral sites (ω = 1) and positive sites (ω > 1), respectively
